# Cement for Wood and Iron

**Published:** 1879-04

**Authors:** 


CEMENT FOR WOOD AND IRON.—A cement made of oxide of lead and concentrated glycerine unites wood to iron with remarkable efficiency. The composition is insoluble in acids, is unaffected by the action of moderate heat, sets rapidly, and acquires an extraordinary hardness.—*Louisville Med. News.*

The medical schools of the United States, according to Prof. Pepper, number sixty-five, without counting the schools of homœopathy, eclectics, etc. Of these there are fifteen which are useful practically. The other fifty are not only useless, but inaccessible to all improvement, and only fit to be suppressed. Less time is required to study medicine in American Medical Colleges than to study horse diseases in the Parisian Veterinary Colleges. This defective system of American schools results, first, in increasing outrageously the number of doctors in America one to every six hundred of the population, while in France there is only one to every two thousand persons; and, secondly, in increasing degradation of medical pursuits. This is a grave state of things which the brilliant American surgeons, though rare exceptions, ought to know and fight against.—*Va. Med. Monthly.*

